# Damp Heat Treatment of Cu(In,Ga)Se_2_ Solar Cells with Different Sodium Content

**DOI:** 10.3390/ma6125478

**Published:** 2013-11-27

**Authors:** Felix Daume, Stefan Puttnins, Christian Scheit, Hendrik Zachmann, Andreas Rahm, Alexander Braun, Marius Grundmann

**Affiliations:** 1 Solarion AG, Ostende 5, Leipzig 04288, Germany; E-Mails: stefan.puttnins@solarion.de (S.P.); christian.scheit@solarion.de (C.S.); hendrik.zachmann@solarion.de (H.Z.); andreas.rahm@solarion.de (A.R.); alexander.braun@solarion.de (A.B.); 2 Institut für Experimentelle Physik II, Universität Leipzig, Linnéstr. 5, Leipzig 04103, Germany; E-Mail: grundmann@physik.uni-leipzig.de

**Keywords:** CIGSe, long-term stability, degradation, damp heat, sodium

## Abstract

Long term stability is crucial to maturing any photovoltaic technology. We have studied the influence of sodium, which plays a key role in optimizing the performance of Cu(In,Ga)Se${}_{2}$ (CIGSe) solar cells, on the long-term stability of flexible CIGSe solar cells on polyimide foil. The standardized procedure of damp heat exposure (85% relative humidity at 85 ${}^{\circ }$C) was used to simulate aging of the unencapsulated cells in multiple time steps while they were characterized by current-voltage analysis, capacitance-voltage profiling, as well as electroluminescence imaging. By comparing the aging process to cells that were exposed to heat only, it could be confirmed that moisture plays the key role in the degradation process. We found that cells with higher sodium content suffer from a more pronounced degradation. Furthermore, the experimental results indicate the superposition of an enhancing and a deteriorating mechanism during the aging process. We propose an explanation based on the corrosion of the planar contacts of the solar cell.

## 1. Introduction

Solar cells based on chalcopyrite Cu(In,Ga)Se${}_{2}$ (CIGSe) absorbers are currently entering mass market production volumes (e.g., a gigawatt production capacity was hit by Solar Frontier [1]) as they became competitive with polycrystalline silicon solar cells in terms of efficiency, reaching up to 20.3% on glass [2] and 20.4% on polyimide [3] substrate. Delivering CIGSe on polyimide combines the general advantages of thin film solar cells, such as the economic use of raw materials, lower energy consumption for production and, hence, a lower energy payback time [4] with the specific features of a lightweight flexible substrate, *i.e*., higher specific power, the feasibility for roll-to-roll processing and the ability to manufacture a flexible module, paving the way for new applications and markets (e.g., applications on curved surfaces or installation on low-load rooftops).

As the CIGSe technology moves from lab-scale fabrication to a profitable production on an industrial scale, long-term stability is crucial. In order to maintain high conversion efficiency long term, an in-depth understanding of the specific processes of degradation for each component of a solar module, and, particularly, the solar cell with its complex stack of functional layers, is indispensable. A standardized and widely accepted procedure to simulate the aging of modules or cells is the exposure to damp heat (85% relative humidity at 85 ${}^{\circ }$C), proving the excellent stability for flexible modules of Solarion [5]. For CIGSe, it is known that sodium in the absorber plays a key role in optimizing the performance of the solar cell [6,7]. An improved morphology, a higher conductivity and higher p-doping, respectively, as well as an increased open circuit voltage are among the discussed beneficial effects of sodium. An influence of sodium on the degradation of CIGSe solar cells covered with silicon nitride barrier films was reported [8], which was facilitated by sodium migration in the cell. However, little seems to be known about the influence of sodium on the properties of the bare device over time.

In this study, we examine unencapsulated CIGSe solar cells with different sodium content with respect to their aging behavior. The cells are consecutively exposed to damp heat and characterized by current-voltage analysis, capacitance-voltage profiling, as well as electroluminescence imaging, providing a time-resolved perspective on the aging process. The combination of these techniques allows one to reveal and interpret the correlation of the aging behavior of CIGSe solar cells with the sodium content, as well as the identification of a main mechanism of degradation.

## 2. Experimental

CIGSe-based flexible solar cells at Solarion AG are produced in a unique ion beam-assisted deposition process [9,10]. In a roll-to-roll setup, the flexible polyimide substrate is sputter coated with a molybdenum back contact. Subsequently the CIGSe absorber is deposited via co-evaporation of Cu, In and Ga and the use of a Se ion beam. Sodium is provided via co-evaporation from a NaF source during CIGSe deposition. A CdS buffer layer is deposited in a wet chemical process; then, the window layer (i-ZnO, ZnO:Al) is sputter deposited. Finally individual cells are cut out of the web, and a metallic grid is screen printed on top of the cells.

For this study, 18 solar cells of the size of 126 × 31 mm${}^{2}$ were chosen out of a modified manufacturing run. The set of samples consisted of two groups each with a different sodium content (“A”, “B”), which was set by tuning the evaporator temperature, $T_{NaF}$, of the sodium source (NaF) during CIGSe deposition in two steps (A: $T_{NaF}=775{\;}^{\circ }$C, B: $T_{NaF}=825{\;}^{\circ }$C). With the higher temperature of the NaF source, more sodium is supplied and incorporated into the absorber [7]. Six cells of each group were exposed to “damp heat” (85 ${}^{\circ }$C at 85% relative humidity), whereas three cells were exposed to “dry heat” (85 ${}^{\circ }$C in a convection oven).

All samples were bonded to a rigid glass carrier to ensure safe and damage-free handling. After each aging cycle, all cells were characterized via current-voltage (IV), capacitance-voltage (CV) measurements and electroluminescence (EL) imaging. This cyclic approach of the experiment allows not only for the observation of the potentially different degradation behavior of different cells, it also allows a time-resolved insight into the progression of degradation. In order to center statistical variations between the samples, the results presented in this paper are the averaged data for each group (six or three cells, respectively).

The IV measurements were carried out after 1 h of light soaking (approximately 100 W/m${}^{2}$) under a steady-state solar simulator providing standard test conditions (AM1.5 spectrum, 1000 W/m${}^{2}$, 25 ${}^{\circ }$C sample temperature). IV data in the range of −0.3 V to 1.2 V were recorded with a Keithley 2601A source meter, analyzed in order to obtain the open circuit voltage, $V_{OC}$, the short circuit current density, $J_{SC}$, the fill factor, FF, the efficiency, *η*, the series resistance, $R_{S}$, as well as the shunt (or parallel) resistance, $R_{P}$, and fitted with a non-ideal one-diode model (equivalent circuit: diode in parallel with a shunt resistor, both of which are in series with another resistor), providing the diode ideality factor, *n*, and the saturation current density, $J_{0}$:

(1) $$J\left(V\right)=J_{0}\left(\exp\left(\frac{e(V-JR_{S})}{nkT}\right)-1\right)+\frac{V-JR_{S}}{R_{P}}-J_{Ph}$$

(where *e* is the elementary charge, *T* the temperature and $J_{Ph}$ the photo current density). All CV measurements were carried out with an Agilent 4284A impedance analyzer at room temperature in the dark. The capacitances were measured at 10 kHz with voltage biases ranging from 0.3 V to −1 V and an AC oscillation amplitude of 50 mV. From the capacitance measured at reverse bias voltages, a profile of the net doping, $N_{d}$, could be derived [11] under the common assumption of ϵ=10 for the dielectric constant [12]. All net dopings presented in this paper refer to the value obtained this way at zero bias voltage.

The electroluminescence (EL) images were captured with a Sensovation coolSamBa HR-830 for the near-infrared operating at a resolution of 1,108 by 834 pixels, while the cell was supplied with a current density of 26.7 mA/cm${}^{2}$.

## 3. Results and Discussion

There was no damage visible on the cells after damp heat. High-resolution electron microscopy images (cross-section and top view) of similar samples showed no differences either. Thus, the damp heat treatment up to 50 h discussed in this paper led to mild damage, *i.e*., no physical damage, such as delamination, but degradation in the electrical (and optical) properties.

For all cells exposed to damp heat, we observed changes in performance over time. Usually, the treatment has a detrimental influence on the performance. It is the main objective of this paper to contribute to the understanding where (in the cell) these changes result from and how certain parameters, namely sodium content, influence the degradation behavior. However, in order to interpret the data, it is necessary to distinguish between the influences of moisture (“damp heat”) and heat (“dry heat”).

### 3.1. Damp and Dry Heat

**Figure 1:**
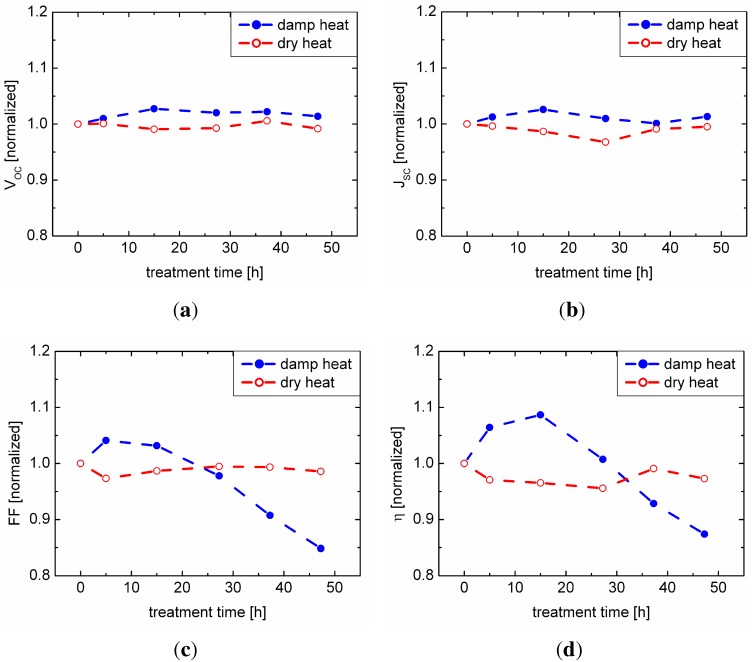
Comparison of damp and dry heat treatment of the set of samples with sodium content A; (a) open circuit voltage; (b) short circuit current density; (c) fill factor; (d) efficiency. All values are scaled to the initial (0 h) value.

In Figure 1, the results from the IV measurements of samples with sodium content A are shown comparing damp and dry heat treatment. The set of samples exposed to dry heat shows no significant aging effect in any of the IV parameters, whereas the set of samples exposed to damp heat shows a degradation behavior. While the open circuit voltage and the short circuit current remain unaffected, mainly the fill factor and the efficiency are affected.

Regarding the damp heat treatment, the diode-related internal parameter, $V_{OC}$, as a measure for the CIGSe/CdS junction quality and $J_{SC}$, being related to carrier collection, do not significantly change. Thus, the observations from Figure 1 suggest that the performance after aging is limited by a decreasing fill factor caused by the detrimental influence of an increasing series resistance. The $R_{S}$ values determined from the IV characteristics confirm an increase by a factor of 1.4 (4.2) for the samples with sodium content A (B). Since this change can only be observed for samples exposed to damp heat, we conclude that moisture plays a key role in the degradation process.

In contrast to the overall degradation of the damp heat samples, we observe an intermediate increase in fill factor and efficiency after 5 h and 15 h of damp heat treatment, hinting at a superposition of an enhancing and a deteriorating mechanism involved in the aging process.

### 3.2. Electrical Performance

Since we have confirmed damp heat as a suitable technique for the simulation of aging, in the following, we will only discuss data from the sets of samples that were exposed to damp heat. None of the IV parameters of the samples exposed to dry heat changed beyond the level of confidence of the measurement.

**Figure 2:**
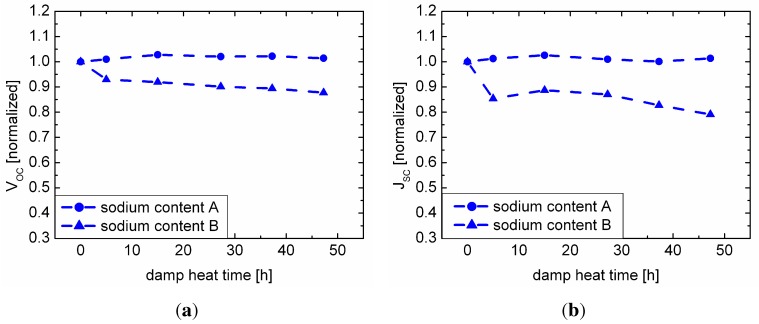

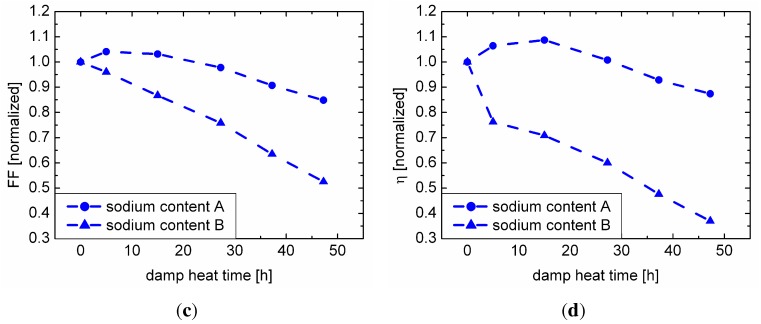
Damp heat treatment of the set of samples with sodium content A and B; (a) open circuit voltage; (b) short circuit current density; (c) fill factor; (d) efficiency. All values are scaled to the initial (0 h) value.

Time-resolved data on the degradation behavior of the samples with respect to the sodium content in the CIGSe absorber layer are presented in Figure 2. Both sets of samples show a decrease of efficiency and fill factor under exposure to damp heat. However, only for the cells with lower sodium content A did we observe a slight increase in efficiency and fill factor before the overall decline dominates. As mentioned in Section 3.1, this suggests that the degradation is superimposed by an enhancing mechanism, initially. Its origin is not understood, yet. The degradation is much more pronounced for the samples with higher sodium content B. While visible for lower amounts of sodium in the sample, the enhancing mechanism is suppressed at this higher sodium content; thus, the degradation dominates for all damp heat time steps. As we have seen before, for the samples with sodium content A, the open circuit voltage and the short circuit current density do not decline. For the samples with sodium content B, the same is true, except for the dip after 5 h of damp heat.

### 3.3. Spatial Effect on Electroluminescence

**Figure 3:**
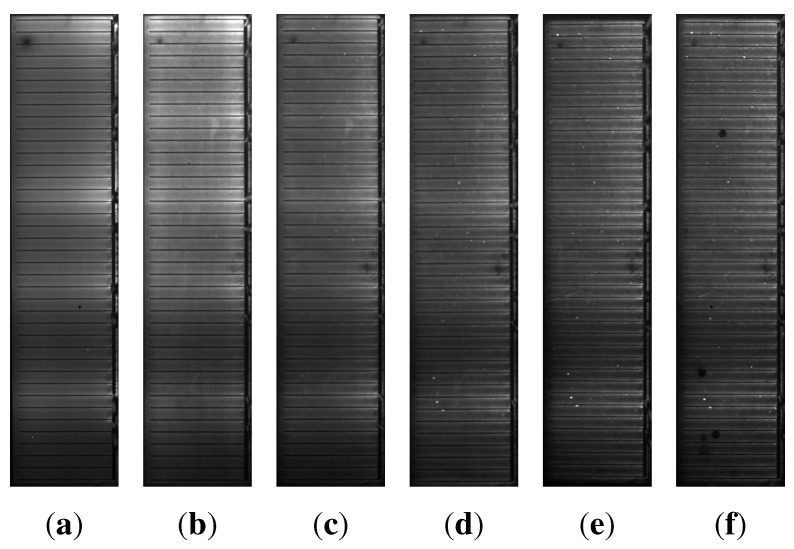
Electroluminescence images of one of the samples with sodium content A after a damp heat treatment of (a) 0 h; (b) 5 h; (c) 15 h; (d) 27 h; (e) 37 h; (f) 47 h.

**Figure 4:**
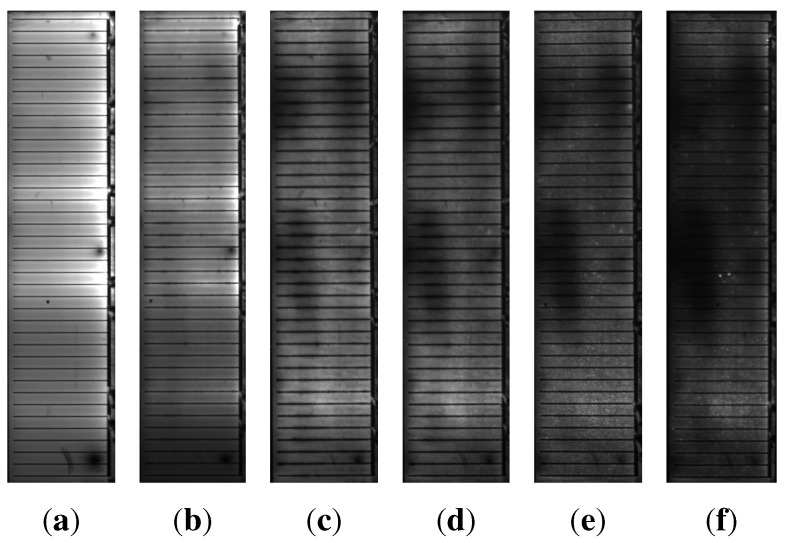
Electroluminescence images of one of the samples with sodium content B after a damp heat treatment of (a) 0 h; (b) 5 h; (c) 15 h; (d) 27 h; (e) 37 h; (f) 47 h.

The intensity of the EL signal of all samples under damp heat treatment decreases with time, whereas it remains unaffected for the samples under dry heat. The decrease in EL intensity is much more pronounced for the samples with higher sodium content B (Figure 4) than for the samples with sodium content A (Figure 3). For the samples with sodium content B, an effect can be observed that is barely visible for the samples with sodium content A: the formation of darker cloud-like areas within the cell (Figure 4).

### 3.4. Capacitance and Doping

**Figure 5:**
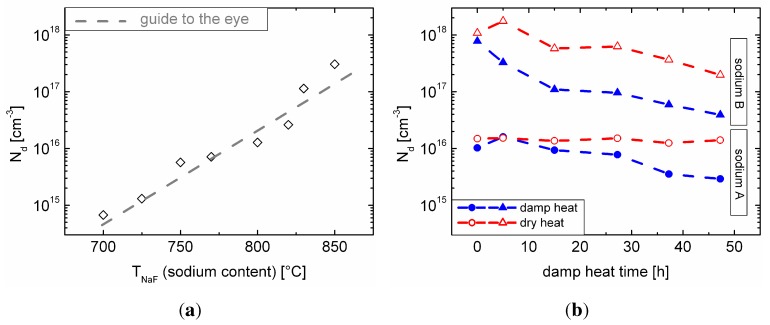
(a) Net doping, $N_{d}$, of the absorber in dependence of the sodium content in the Cu(In,Ga)Se${}_{2}$ (CIGSe) layer; (b) change of the net doping, $N_{d}$, of the sets of samples with sodium content A and B under damp and dry heat treatment.

The derivation of net doping profiles from CV measurements usually contributes to the understanding of the effects of sodium on the CIGSe absorber. In Figure 5(a) the influence of the sodium content on the net doping, $N_{d}$, of the absorber (before damp heat), derived from the measurement of more than 150 cells in total (from the same manufacturing run as the other samples), is depicted. With increasing sodium content, the net doping increases. Described by many authors, this is commonly attributed to a decrease of the degree of compensation in the CIGSe absorber (for instance, since sodium suppresses donor type $In_{Cu}$ antisites [6]).

**Figure 6:**
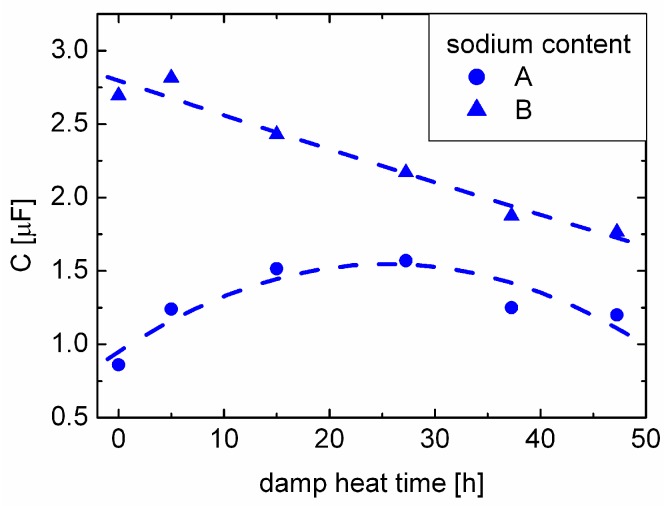
Capacitances of the sets of samples with sodium content A and B *versus* time in damp heat.

CV measurements were carried out after each damp heat aging step, as well. The trend of the capacitance (at zero bias) under damp heat treatment is plotted in Figure 6. We will use this representation of the CV data in Section 3.5 to explain the degradation behavior of our solar cells. Additionally, the net doping of the absorber was derived from the CV data (Figure 5(b)). In accordance with Figure 5(a), the samples with sodium content B exhibit a higher net doping, as more sodium was supplied during CIGSe deposition. Apparently, for the samples with sodium content A, the net doping remains unaffected under dry heat treatment, while there is a slight decrease for the samples with sodium content B. Exposure to damp heat significantly decreases the apparent net doping for both sets of samples; thereby, this effect is much more pronounced for the samples with higher sodium content (samples of B).

### 3.5. Discussion and Interpretation

First, we try to explain the trend in the capacitance data in terms of doping, but motivated by the contradiction of this interpretation with the IV data, we then propose a new explanation.

The more pronounced decrease of the net doping with higher sodium content (Figure 5(b)) fits well with the more pronounced degradation of the whole cell in terms of fill factor and efficiency (Figure 2). As a simple model, one could imagine that sodium is “washed out” of the cell by the water under damp heat conditions. Thus, degradation is solely visible (and sodium-dependent) in the presence of water, whereas in dry heat, the samples remain unaffected. One may then speculate that the decrease in net doping for samples with sodium content B under dry heat treatment (Figure 5(b)) is explained by the influence of ambient air humidity during measurement, due to the hygroscopic nature of compounds, like sodium selenide. When the sodium content decreases, the degree of compensation in the material increases again, thereby having a detrimental effect on the net doping (Figure 5(a)). The CV measurements showed that along with the decrease of net doping, the width of the space charge region increased. If one assumes a fixed charge carrier diffusion length, the short circuit current density should increase along with the width of the space charge region under damp heat treatment, due to a better carrier collection in the absorber.

Nevertheless, $J_{SC}$ remains nearly constant. Dropping the assumption of a fixed diffusion length (which is rather unlikely, since the carrier lifetime in CIGSe decreases already under atmospheric conditions [13]), the increase in the space charge region width had to compensate for the decrease in diffusion length completely in order to leave $J_{SC}$ unaffected. This rather unlikely relationship led us to look for an alternative explanation of all collected data. Moreover, the apparent decrease in net doping for the damp heat treated solar cells contradicts their stable open circuit voltages.

The standard interpretation of the capacitance data is based on strong simplifications of the device: with the impedance analyzer, we measure the complex resistance of the sample. Under the assumption of a parallel equivalent circuit of a capacitor (formed by the space charge region of the p-n junction) and a shunt resistance, we obtain a capacitance value. According to the again simplified interpretation as a plain plate-type capacitor, the capacitance, *C*, depends on the distance of the plates (*i.e*., the width of the space charge region, *w*) and the area, *A*, of the capacitor (*i.e*., the area of the p-n junction):

(2) $$C=\epsilon \epsilon_{0}A/w$$

(where *ϵ* is the dielectric constant of the CIGSe absorber and $\epsilon_{0}$ the vacuum permittivity). Since not only the junction, but also the front and back contacts of the solar cell extend over this area, it is likely that the non-idealities of these contacts have an influence on the measurement.

We therefore propose an explanation of all measured data based on the corrosion of the planar contacts. Sodium in the CIGSe solar cell in conjunction with water leads to the corrosion of both planar contacts: the ZnO:Al window layer and the molybdenum back contact. However, it seems more likely that the latter process and, especially, the degradation of the molybdenum-CIGSe interface is responsible for the sodium-related degradation of the solar cells, since it is known from elemental depth profiling (secondary ion mass spectrometry (SIMS)) that sodium tends to aggregate at the back contact interface [14,15]. With SIMS measurements on a similar set of samples, we found a decrease of the sodium aggregation at the molybdenum-CIGSe interface after damp heat treatment [15]. Since water is necessary for the described corrosion mechanism, we observe it in the samples treated with damp heat, but not in the samples treated with dry heat. As the polyimide substrate is permeable to water [16], the ingress from the back side of the cell is another possible path for the water to enter the cell besides the top (window layer) surface. The columnar structure of the molybdenum layer (resulting from the columnar growth during the sputter process) might even provide capillary-like channels for the water to propagate.

Literature suggests that only well-defined molybdenum-CIGSe interfaces with an intermediate MoSe${}_{2}$ layer [17] result in good ohmic contacts [18]. A partially corroded back-contact (interface) will then presumably exhibit a significantly increased (contact) resistance. The partial corrosion of the back contact interface then results in a lower effective interface area, *A*, between molybdenum and CIGSe. Looking from this perspective, the changes in the net doping in Figure 5(b) are an artifact from the reduced area, *A*, of the model capacitor. Figure 6 shows the change in capacitance of the two sets of samples under damp heat treatment. The samples show a decrease of the capacitance with damp heat time, consistent with our explanation. However, for the samples with sodium content A, the capacitance shows an initial increase consistent with the observation of an initially increasing efficiency. This effect could be related to water ingress into the CIGSe absorber, possibly having a positive influence on the efficiency, similar to the one of water vapor during CIGSe growth [19]. Therefore, damp heat exposure could lead to a superposition of the enhancing effect on the absorber and the detrimental effect on the back contact, resulting in the efficiency (Figure 2(d)) and capacitance maxima (Figure 6) for the samples with sodium content A. For the samples with sodium content B, the back contact corrosion seems to be dominant; hence, no initial increase in efficiency or capacitance can be observed.

The idea of a dominating back contact corrosion also explains the EL data better than the reduced net doping, which should affect the absorber and, therefore, the EL signal uniformly over the whole area, *A*. Certainly that would be in contradiction to the observation of localized darker areas arising in the EL images under damp heat treatment (Figure 4). On the other hand, since the occurrence of corrosion will most likely be dominated by inhomogeneities of the water ingress resulting from, e.g., percolation behavior at grain boundaries or pinholes in the layer stack and inhomogeneities in the initial sodium distribution, the more pronounced corrosion in certain areas is consistent with our explanation. Quantitative support for our explanation is provided by the comparison of the effects of damp heat on the capacitance and the EL signal. An image analysis (measuring the two disjoint areas after defining a brightness threshold in Figure 4(f)) yields that approximately 20% of the cell area belongs to dark spots. This estimation is rough, since it is difficult to define an appropriate threshold. However, the fraction of dark spots is well within the same order of magnitude as the change in capacitance of approximately 40% for the set of samples with sodium content B.

Although the effect of the back contact corrosion seems to be the main degradation mechanism in the long run for the samples in this study, our explanation does not rule out other degradation mechanisms related to the absorber itself or one of the various other layers and interfaces. Other aging mechanisms might even be present in our experiment (as hinted at by the intermediate increase in efficiency for the samples with sodium content A), but they are superimposed by the described corrosion effect.

## 4. Conclusions

Within our study, dry heat treatment had no significant detrimental influence on unencapsulated CIGSe solar cells on flexible polyimide substrates. In contrast, damp heat treatment diminished the performance of the cells. Samples with higher sodium content showed a stronger degradation behavior in terms of fill factor, efficiency and EL intensity than those with lower sodium content. We presented a strong indication that the main degradation path is the corrosion of the back contact (interface); however, a slight increase of the performance after short damp heat treatment indicates the presence of another process.

The finding that sodium influences the long-term stability of CIGSe solar cells necessitates precise metering of the amount of sodium provided during future absorber deposition in order to achieve an acceptable long-term stability of the device besides an optimized initial performance.

## References

[1] Japan’s Largest Solar Panel Factory Reaches Full Commercial Operations, Announces Solar Frontier. http://www.solar-frontier.com/eng/news/2011/C002132.html.

[2] Jackson P., Hariskos D., Lotter E., Paetel S., Wuerz R., Menner R., Wischmann W., Powalla M. (2011). New world record efficiency for Cu(In,Ga)Se_2_ thin-film solar cells beyond 20%. Progr. Photovolt. Res. Appl..

[3] Neuer Weltrekord für den Wirkungsgrad von Solarzellen. http://www.empa.ch/plugin/template/empa/*/131475/---/l=1.

[4] Alsema E.A., de Wild-Scholten M.J., Fthenakis V.M. Environmental Impacts of PV Electricity Generation—A Critical Comparison of Energy Supply Options. Proceedings of the 21st European Photovoltaic Solar Energy Conference.

[5] Reithe A., Wachsmuth M., Münch M., Meiner M., Tegen S., Heger K. From Rigid to Flexible: The Real Challenge of CIGS Module R&D. Proceedings of the PV Module Reliability Workshop.

[6] Rau U., Schock H.W. (1999). Electronic properties of CIGS heterojunction solar cells—Recent achievements, current understanding, and future challenges. Appl. Phys. A Mater. Sci. Process..

[7] Zachmann H., Puttnins S., Daume F., Rahm A., Otte K., Caballero R., Kaufmann C.A., Eisenbarth T., Schock H.W. (2010). Incorporation of Na in low-temperature deposition of CIGS flexible solar cells. Mater. Res. Soc. Proc..

[8] Elowe P.R., Stempki M.A., Rozeveld S.J., DeGroot M.W. (2011). Development of direct cell inorganic barrier film technology providing exceptional device stability for CIGS solar cells. Chem. Mater..

[9] Otte K., Makhova L., Braun A., Konovalov I. (2006). Flexible Cu(In,Ga)Se_2_ thin-film solar cells for space application. Thin Solid Films.

[10] Zachmann H., Puttnins S., Daume F., Rahm A., Otte K. (2011). Generation of electrical defects in ion beam assisted deposition of Cu(In,Ga)Se_2_ thin film solar cells. Thin Solid Films.

[11] Hegedus S.S., Shafarman W.N. (2004). Thin-film solar cells: Device measurements and analysis. Progr. Photovolt. Res. Appl..

[12] Rau U., Braunger D., Herberholz R., Schock H.W., Guillemoles J.F., Kronik L., Cahen D. (1999). Oxygenation and air-annealing effects on the electronic properties of CIGS films and devices. J. Appl. Phys..

[13] Metzger W.K., Repins I.L., Romero M., Dippo P., Contreras M., Noufi R., Levi D. (2009). Recombination kinetics and stability in polycrystalline Cu(In,Ga)Se_2_ solar cells. Thin Solid Films.

[14] Cesar J., Puttnins S., Daume F., Braun A., Rahm A. Temperature-Dependent IV and EQE Measurements on CIGSe Solar Cells with Varying Sodium Content. Proceedings of the 27th European PV Solar Energy Conference.

[15] Daume F., Rahm A., Braun A., Grundmann M. Sodium in the Degradation Process of Cu(In,Ga)Se_2_ Solar Cells. Proceedings of the 28th European PV Solar Energy Conference.

[16] McClure D.J. (2010). Polyimide film as a vacuum coating substrate. Soc. Vac. Coaters.

[17] Abou-Ras D., Mukherji D., Kostorz G., Brémaud D., Kälin M., Rudmann D., Döbeli M., Tiwari A.N. (2005). Dependence of the MoSe_2_ formation on the Mo orientation and the Na concentration for Cu(In,Ga)Se_2_ thin-film solar cells. Mater. Res. Soc. Proc..

[18] Wada T., Kohara N., Nishiwaki S., Negami T. (2001). Characterization of the CIGS/Mo interface in CIGS solar cells. Thin Solid Films.

[19] Ishizuka S., Sakurai K., Yamada A., Matsubara K., Shibata H., Yonemura M., Nakamura S., Nakanishi H., Kojima T., Niki S. Water Vapor Introduction During Cu(In_1-x_Ga_x_)Se_2_ Thin-Film Deposition and its Effect on Solar Cell Performance. Conference Record of the IEEE 4th World Conference on Photovoltaic Energy Conversion.

